# A Diagnostic Pitfall: Chronic Schistosomiasis Presenting With Inflammatory Bowel Disease-Like Symptoms in an Endemic Area

**DOI:** 10.7759/cureus.93422

**Published:** 2025-09-28

**Authors:** Afraa Mohammed, Hala Abdalla, Alaa Mohamed, Elhadi Awooda

**Affiliations:** 1 Gastroenterology and Hepatology, Sudan National Center for Gastrointestinal and Liver Diseases, Khartoum, SDN; 2 Histopathology, Sudan National Center for Gastrointestinal and Liver Diseases, Khartoum, SDN; 3 Research and Development, Sudan National Center for Gastrointestinal and Liver Diseases, Khartoum, SDN

**Keywords:** eosinophilia, healthcare in sudan, inflammatory bowel disease, intestinal schistosomiasis, ova, schistosomiasis

## Abstract

A 24-year-old male presented with a two-year history of intermittent bloody diarrhoea and central abdominal pain that had gradually worsened, accompanied by joint discomfort and backache. He denied direct exposure to irrigation canals, drains, or freshwater bodies usually linked to schistosomiasis transmission. However, he recalled occasional visits to the Nile Corniche where his feet had contact with fresh water. Laboratory investigations revealed marked eosinophilia (14%), which, in combination with intestinal and extra-intestinal features, raised suspicion for inflammatory bowel disease. Colonoscopy showed diffuse reddish patches with mild exudation but lacked a conclusive diagnosis. Histopathological analysis confirmed chronic schistosomiasis through the identification of viable *Schistosoma* ova, sessile polyps, and eosinophilic granulomas. The patient received 2400 mg of praziquantel and experienced complete resolution of symptoms.

## Introduction

Schistosomiasis is a parasitic infection of considerable global health significance, particularly in tropical and subtropical regions where access to clean water is limited [1]. More than 200 million individuals are affected worldwide, primarily due to infection with blood flukes of the genus *Schistosoma* [1]. The disease is endemic in 78 countries, with most cases occurring in sub-Saharan Africa, including parts of the Eastern Mediterranean region [2]. In Sudan, schistosomiasis is common in irrigation zones such as Gezira, the North Province (Wadi Halfa, Dongola, Berber), Blue Nile State, and areas of Kordofan and Darfur [3]. Women and children are particularly at risk [2]. According to the WHO, 251.4 million people required preventive therapy in 2021 [4].

Chronic intestinal schistosomiasis is associated with infection by *S. mansoni*, *S. japonicum*, *S. intercalatum*, *S. mekongi*, and occasionally *S. haematobium*. The disease course is influenced by frequency of exposure and parasite burden in the colonic and rectal mucosa, leading to inflammation, granuloma formation, fibrosis, and ulceration. Symptoms include anorexia, weight loss, asthenia, and diarrhoea, with severe cases resulting in anaemia due to mucosal bleeding. The condition can mimic inflammatory bowel disease (IBD) or colorectal cancer, and predispose to strictures and polyp formation [5].

The immune response to *Schistosoma* involves macrophage activation. Th1 responses promote microbial destruction at the expense of tissue integrity, while Th2 responses trigger alternatively activated macrophages (M2 or AAMs) via IL-4 and IL-13, which reduce Th1-mediated injury [6]. Schistosomiasis offers a model to explore these immune mechanisms, as *Schistosoma* eggs stimulate granulomatous reactions and a Th2-dominant immune profile, which limits acute mortality but contributes to chronic fibrosis and long-term complications [6,7].

In intestinal cases, *Schistosoma* organisms tend to localise within the venous drainage of the inferior mesenteric plexus, causing submucosal fibrosis and mucosal hyperplasia. Endoscopic evaluation often reveals oedematous mucosa, petechial haemorrhages, nodules, or polyps resembling inflammatory colitis or pseudomembranous colitis [8].

## Case presentation

A 24-year-old male, without prior significant medical history, reported a two-year duration of intermittent abdominal discomfort, bloating, and episodes of bloody diarrhoea mixed with mucus, associated with tenesmus. Over time, the diarrhoea worsened and became nocturnal. He also described persistent joint pains and backache. There was no history of fever, weight change, alcohol use, smoking, or family gastrointestinal disorders. Although he never visited known endemic regions, he had engaged in recreational water contact at the River Nile Corniche. His general examination was normal. The patient had previously been treated with several antibiotic courses for presumed dysentery, without improvement. Due to ongoing bowel and systemic symptoms, IBD was suspected. Several blood tests were performed, which showed an increase in eosinophilia, erythrocyte sedimentation rate (ESR), and C-reactive protein (CRP), as mentioned in Table 1.

**Table 1:** Laboratory and blood investigations. WBC: white blood cells; ESR: erythrocyte sedimentation rate; CRP: C-reactive protein.

Test name	Test result	Normal range
Eosinophilia	14%	Less than 5%
Haemoglobin	13.8 g/dl	13.5 to 17.5 g/dl
Platelets	360 × 10⁹/L	150 to 450 × 10^9/L
WBC	6.6 × 10⁹/L	4.5 to 11.0 x 10^9/L
ESR	36 mm/hr	Less than 15 mm/hr
CRP	105 mg/L	Less than 10 mg/l

Investigations

**Figure 1:**
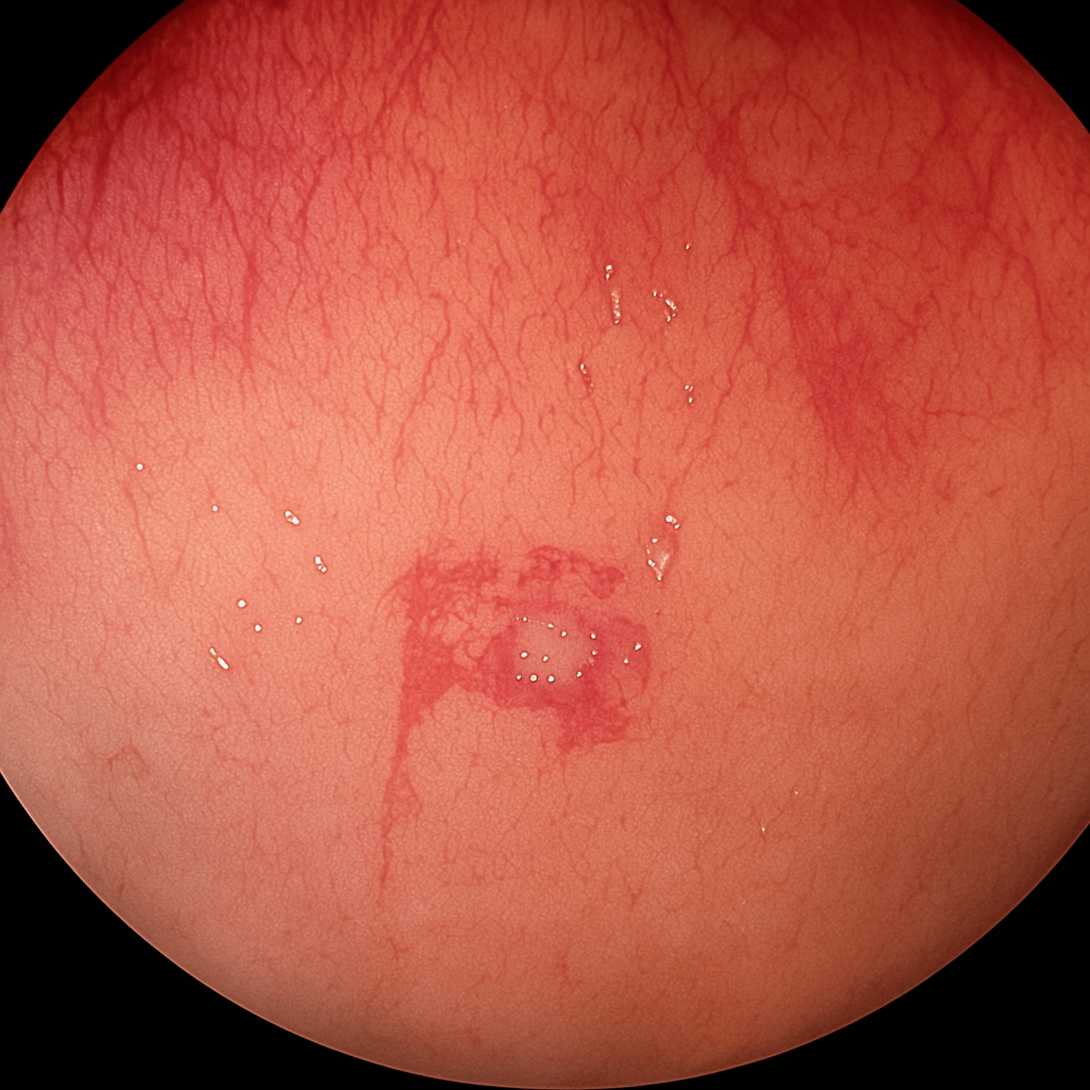
Reddish spots scattered along the left colon.

Viral screening was negative, which excluded other mimicking diseases. Stool microscopy did not detect *Schistosoma* ova. Colonoscopy revealed diffuse reddish spots, mild exudate, and a small sessile polyp (Figure 1).

**Figure 2:**
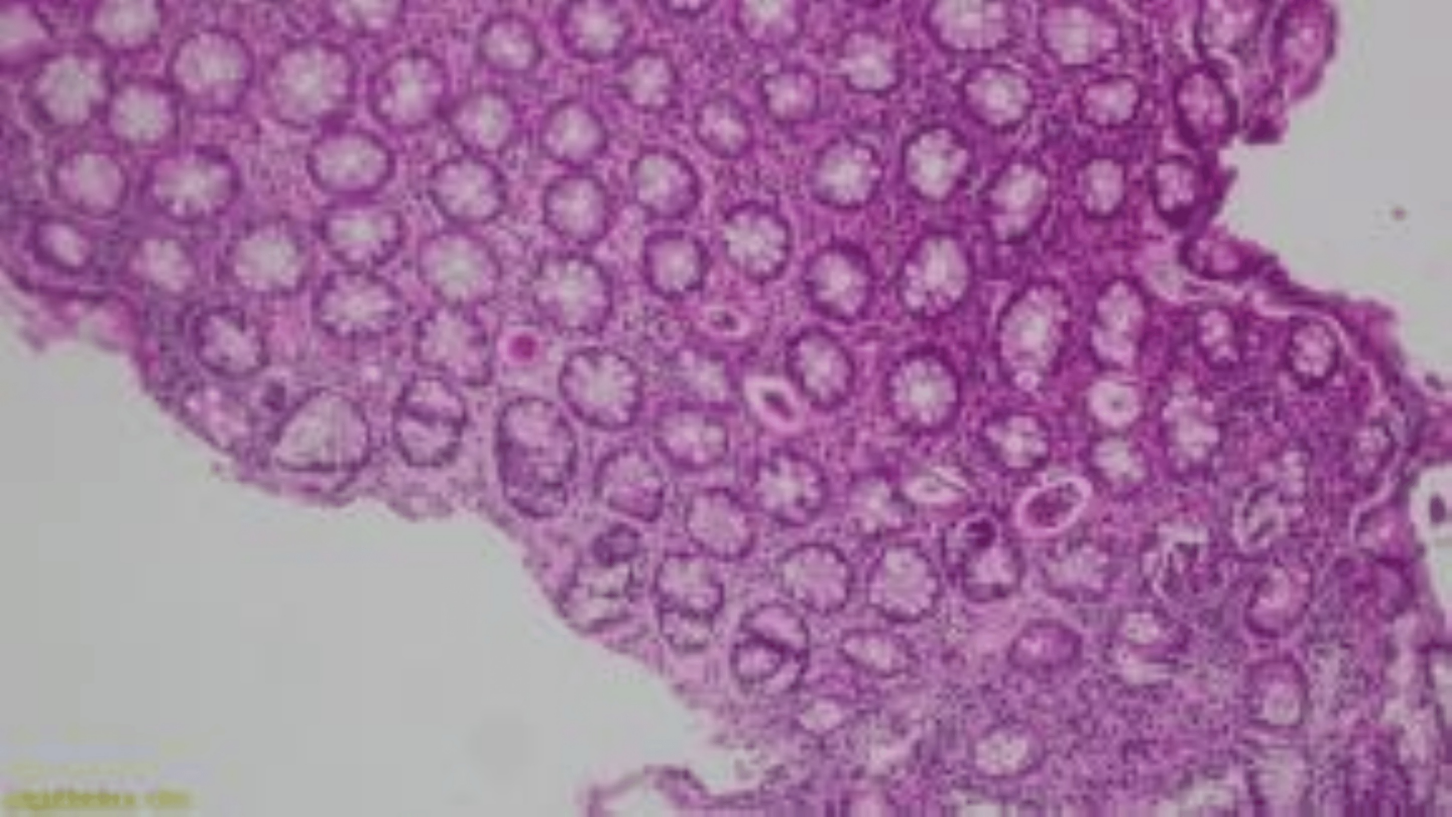
Section from colonic biopsy showing preserved architecture with multiple viable Schistosoma eggs and mixed inflammatory cell infiltrates composed mainly of eosinophils with lymphocytes and plasma cells. Hematoxylin and eosin stain.

**Figure 3:**
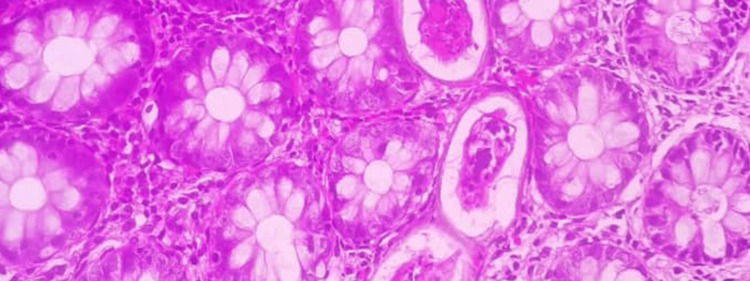
Colonic biopsy showing multiple Schistosoma ova surrounded by granulomatous inflammation and eosinophilic infiltration. Hematoxylin and eosin stain.

**Figure 4:**
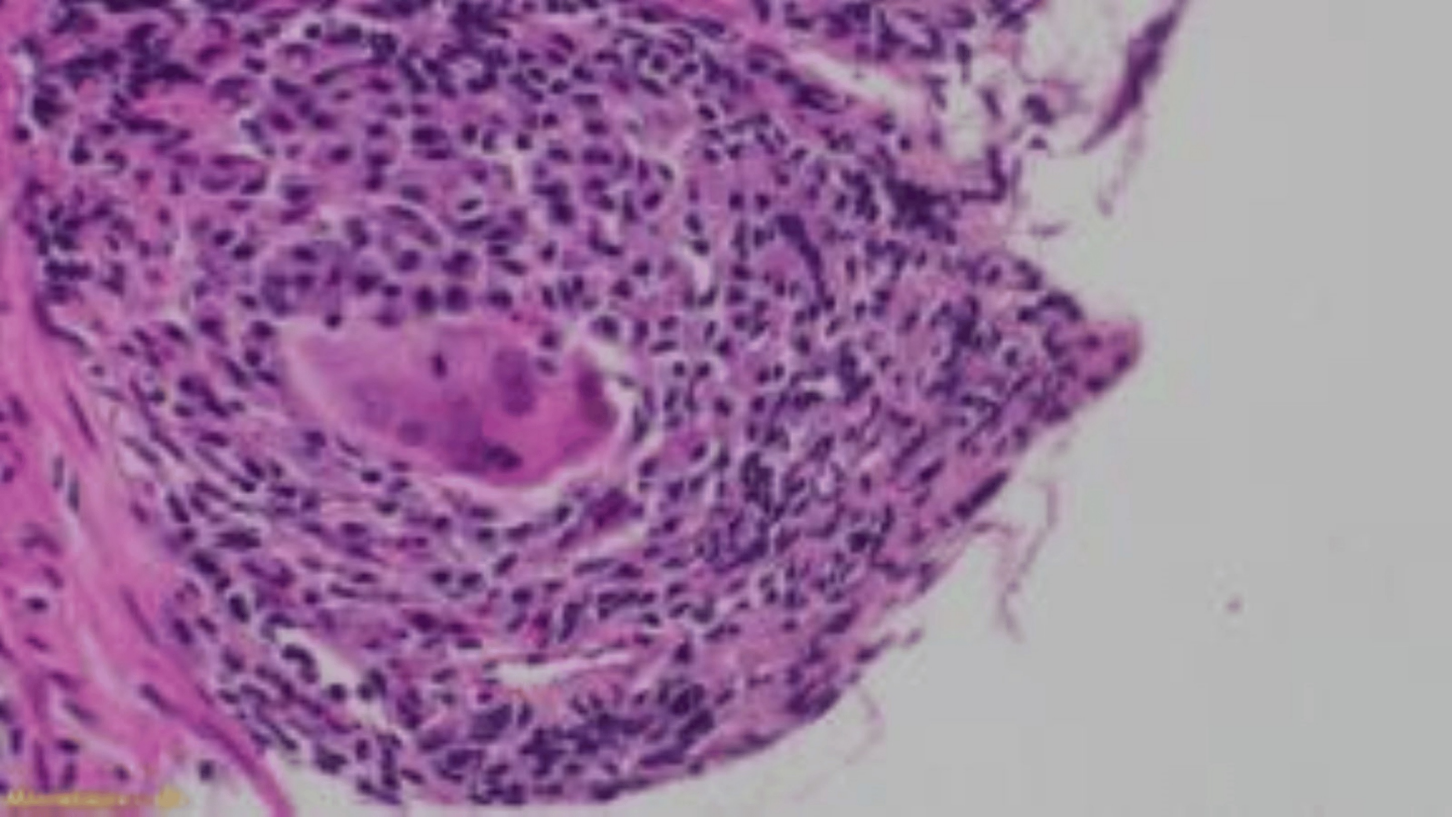
Well-defined granuloma composed of aggregates of histiocytes surrounding Schistosoma eggs.

Multiple biopsies were taken. Histopathology using hematoxylin and eosin stain showed active *Schistosoma* ova, sessile polyps, and inflammatory cell infiltrates (Figures 2, 3), and granuloma formation with preserved crypt architecture (Figure 4).

Differential diagnosis

The main differential diagnosis was IBD, given the patient’s age, clinical course, and endoscopic features. Abdominal tuberculosis was also considered due to its prevalence in Sudan, but the absence of fever, weight loss, and a modest ESR made it less likely to be abdominal tuberculosis. Chronic schistosomiasis was initially thought improbable due to negative stool microscopy and residence outside well-defined endemic zones; however, histopathological confirmation established the diagnosis.

Treatment and outcome

The patient received a single dose of 2400 mg praziquantel, with a repeat dose planned at six months. At follow-ups at one and six months, he reported complete resolution of symptoms and had resumed normal activities. Follow-up and improvement were determined based on symptom resolution.

## Discussion

Schistosomiasis remains a neglected tropical disease with high prevalence in rural African communities, where lack of sanitation and safe water supply perpetuate transmission [3]. Open defecation and urination around water bodies allow eggs to contaminate freshwater, facilitating snail-mediated transmission. In Sudan, canals, drains, and irrigation channels ("abu-eshreen") represent key breeding habitats. Although the River Nile is fast-flowing, stagnant pools along the Corniche may serve as focal transmission sites, as likely occurred in this patient. This case is important because schistosomiasis can mimic chronic IBD, with symptoms such as bloody diarrhoea, mucus, and tenesmus, and also granulomatous inflammation around *Schistosoma* ova demonstrates the chronic immune-mediated tissue damage that underlies many complications of schistosomiasis.

In early stages, ova are readily detected in stool; however, in chronic disease, stool microscopy is frequently negative [9]. In this case, eosinophilia and histological findings of active *Schistosoma* ova within granulomas confirmed chronic schistosomiasis, explaining his long-standing symptoms. Similar cases have been reported globally, including a Guinean male in Turkey, a Filipino woman in Australia, and Ethiopian patients, all initially misdiagnosed as IBD or malignancy before histopathological confirmation [10-12]. Each patient improved following praziquantel therapy.

Experimental studies also suggest schistosomiasis may play a modulatory role in colitis. Deposition of *S. japonicum* eggs protected mice against trinitrobenzenesulfonic acid (TNBS)-induced colitis via immunoregulatory mechanisms involving Th1/Th2 balance and TLR-4 [13]. Liu et al. demonstrated that *S. japonicum* infection prevented dextran sodium sulfate (DSS)-induced colitis by modulating Th1/Th2/Th17 pathways, nuclear factor-κB (NF-κB) signalling, and endoplasmic reticulum stress [14]. Wu et al. showed soluble antigens and recombinant protease inhibitors alleviated colitis by enhancing Treg/Th2 responses and suppressing Th1 responses [15].

A delayed recognition of chronic schistosomiasis can result in complications, including ulceration, strictures, intestinal obstruction, iron-deficiency anaemia, hypoalbuminaemia, and rectal prolapse. Fortunately, praziquantel remains an effective, inexpensive, and well-tolerated treatment. Control strategies must emphasise public health education, safe hygiene, provision of latrines, clean water access, and community interventions, such as infection mapping and mass prophylaxis, which reduce prevalence, limit healthcare burden, and foster socioeconomic progress.

## Conclusions

This case demonstrates how chronic intestinal schistosomiasis can mimic IBD, leading to diagnostic uncertainty and treatment delays. Histopathology was essential in reaching the correct diagnosis, especially with negative stool microscopy. Early recognition and timely treatment with praziquantel are crucial to prevent complications. Comprehensive community-based strategies combining sanitation, education, and mass drug administration remain vital in reducing the schistosomiasis burden in endemic regions.
